# Lead toxicity: a review

**DOI:** 10.1515/intox-2015-0009

**Published:** 2015-06

**Authors:** Ab Latif Wani, Anjum Ara, Jawed Ahmad Usmani

**Affiliations:** 1 Section of Genetics, Department of Zoology, Faculty of Life Science, Aligarh Muslim University, Aligarh, Utter Pradesh, India; 2 Department of Forensic Medicine, Faculty of Medicine, Jawaharlal Nehru Medical College and Hospital, Aligarh Muslim University, Aligarh, Utter Pradesh, India

**Keywords:** lead toxicity, lead poisoning, heavy metals, environmental health

## Abstract

Lead toxicity is an important environmental disease and its effects on the human body are devastating. There is almost no function in the human body which is not affected by lead toxicity. Though in countries like US and Canada the use of lead has been controlled up to a certain extent, it is still used vehemently in the developing countries. This is primarily because lead bears unique physical and chemical properties that make it suitable for a large number of applications for which humans have exploited its benefits from historical times and thus it has become a common environmental pollutant. Lead is highly persistent in the environment and because of its continuous use its levels rise in almost every country, posing serious threats. This article reviews the works listed in the literature with recent updates regarding the toxicity of lead. Focus is also on toxic effects of lead on the renal, reproductive and nervous system. Finally the techniques available for treating lead toxicity are presented with some recent updates.

## Introduction

Lead is the most important toxic heavy element in the environment. Due to its important physico-chemical properties, its use can be retraced to historical times. Globally it is an abundantly distributed, important yet dangerous environmental chemical (Mahaffay, 1990a). Its important properties like softness, malleability, ductility, poor conductibility and resistance to corrosion seem to make difficult to give up its use. Due to its non-biodegradable nature and continuous use, its concentration accumulates in the environment with increasing hazards.

Human exposure to lead and its compounds occurs mostly in lead related occupations with various sources like leaded gasoline, industrial processes such as smelting of lead and its combustion, pottery, boat building, lead based painting, lead containing pipes, battery recycling, grids, arm industry, pigments, printing of books, *etc.*

Though its widespread use has discontinued in many countries of the world, it is still used in many industries like car repair, battery manufacturing and recycling, refining, smelting, *etc.* Lead is a highly poisonous metal affecting almost every organ in the body. Of all the organs, the nervous system is the mostly affected target in lead toxicity, both in children and adults. The toxicity in children is however of a greater impact than in adults. This is because their tissues, internal as well as external, are softer than in adults. Long-term exposure of adults can result in decreased performance in some tests of cognitive performance that measure functions of the nervous system. Infants and young children are especially sensitive to even low levels of lead, which may contribute to behavioural problems, learning deficits and lowered IQ (Rubin & Strayer, 2008). Long-time exposure to lead has been reported to cause anaemia, along with an increase in blood pressure, and that mainly in old and middle aged people. Severe damage to the brain and kidneys, both in adults and children, were found to be linked to exposure to heavy lead levels resulting in death. In pregnant women, high exposure to lead may cause miscarriage. Chronic lead exposure was found to reduce fertility in males (Sokol & Berman, 1991). Blood disorders and damage to the nervous system have a high occurrence in lead toxicity.

## Detection of lead poisoning

Several methods are used to detect elevated blood lead levels. The presence of changes in blood cells visible under the microscope or deletion of dense lines in the bones of children seen on X-ray are signs used for detecting lead poisoning. However the main tool to detect elevated levels of body lead is to measure the level of lead in blood samples. This test gives however only an account of lead present in circulating blood but cannot show how much lead is stored in the body. As of 2012, the Centers for Disease Control and Prevention (USA) have set the standard elevated blood lead level for adults to be 10 μg/dL and for children 5 μg/dL of the whole blood (CDC, 2012). Previously, the standard lead level for children was 10 μg/dL. The appearance of clinical manifestations varies from individual to individual depending on other environmental factors. In some there is a clear appearance of clinical features even at lower levels, while some are asymptomatic even at higher levels of lead present in their body fluids. Children are more prone to the effects of lead because usually their organs are in a developing stage. Thus blood lead levels have to be set lower and must be frequently checked, particularly where contamination is expected.

## Effects of lead poisoning

All along human history, lead poisoning has been reported to have severe effects. Occasional lead poisoning was found to be caused by lead salts used in pottery glazes leached by acidic fruit juices. Beethoven's death has been treated in various reports. Many of them have concluded that he died because of the toxic doses of lead-based treatment administered by his doctor. Analysis of his hair was found to contain elevated levels of lead (Mai, 2006). It is also assumed that in the eighteenth and early nineteenth century lead was illegally added to wine both as a sweetener and to make it appear fresh (Mai, 2006). Lead poisoning is believed to be primarily responsible for the collapse of the Roman Empire, in which lead acetate was used as a sweetener of wine. Its prolonged use was considered to have caused dementia to many Roman emperors. Lead poisoning has also been found to be the cause of anaemia in a number of cases as lead inhibits porphobilinogen synthase and ferrochelatase, preventing both porphobilinogen formation and the incorporation of iron into protoporphyrin IX, which prevents heme synthesis (Cohen *et al*., 1981) or causes ineffective heme synthesis and subsequently microcytic anaemia. One of the mechanisms by which lead interferes with cognition is that it acts as calcium analogue which interferes with ion channels. It has been observed that Pb^2+^ is a potent reversible and selective blocker of voltage-dependent calcium channels at low concentrations (Busselberg *et al*., 1993). In a recent study, the authors showed that the toxic effects on blood cells of rats caused by lead nitrate was alleviated by sodium selenite. They also showed that effects of lead nitrate were more harmful in diabetic than in non-diabetic rats (Bas *et al*., 2015). Oxidative stress was studied by low level lead exposure in first grade Uruguayan children, suggesting its potentially adverse effects on oxidative stress (Roy *et al*., 2015). Impaired respiratory function was observed in workers exposed to lead with elevated blood lead concentration and zinc protoporphyrin concentration (Jurdziak *et al*., 2015).

**Figure 1:**
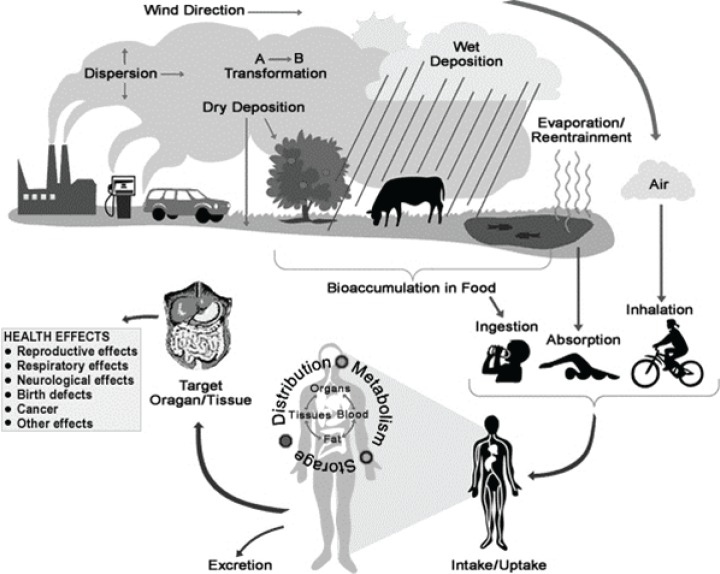
Illustration how people are exposed to chemicals in the environment and the effect of such chemicals on human health.

In a recent study lead and cadmium contents were investigated in counterfeit cigarettes seized in the United States by various law enforcement agencies. Both lead and cadmium levels were found to be markedly higher than in their genuine equivalents. The results suggest the possibility of a higher risk of serious consequences to public health caused by counterfeit cigarettes (He *et al*., 2015).

## Possible pathway of lead poisoning

Poisoning due to lead occurs mainly by ingestion of food or water contaminated with lead. However accidental ingestion of contaminated soil, dust or lead based paint may also result in poisoning. Lead is thought to be quickly absorbed in the blood stream and is believed to have adverse effects on certain organ systems like the central nervous system, the cardiovascular system, kidneys, and the immune system (Bergeson, 2008). Most pharmaceutical companies have set a limit for maximum daily intake of lead as 1.0 μg/g, however prolonged intake of even this low level of lead is hazardous to human beings. Occupational exposure also results in elevated blood lead levels. Increased blood levels are associated with delayed puberty in girls (Schoeters *et al*., 2008). There is no threshold value for the level of lead present in blood below which its concentration can be considered safe. Extremely low yet permanent levels of lead exposure were found to reduce the cognitive capacity of children (Needlemann *et al*., 1990). Dangers from lead poisoning due to paint pigments mainly in children have sharply reduced the use of lead in paints. However old houses may still contain substantial amounts of lead paint (Levin *et al*. 2008). This sometimes causes an accidental contamination of lead in children. In industrialised countries white lead paint has been completely withdrawn from sale, however yellow lead chromate is still in use. Old paint should not be stripped by sanding, as this produces inhalable dust (Marino *et al*., 1990). Traditional medicines were also found to contain heavy metals including lead. A number of diseases have been reported due to consumption of traditional medicine (Karri *et al*., 2008). Ayurvedic medicines are considered to be heavily contaminated with heavy metals. In one recent study the blood lead levels were evaluated in consumers of ayurvedic medicines. Of the 115 participants 40% were found to have an elevated blood lead levels of 10 μg/dL or above and 9.6% had blood lead levels above 50 μg/dL (Breeher *et al*., 2015). Recently a patient taking Chinese folk remedies was reported to suffer from dysplastic changes in erythroid precursors due to lead poisoning (Lv *et al*., 2015).

The Centers for Disease Control and Prevention also issued guidelines about ingesting certain folk medicines which contain a high level of lead and may expose people to lead or lead compounds. For example Daw Tway is a digestive aid used in Thailand and Myanmar (Burma). Analysis of Daw Tway samples showed as much as 970 parts per million (ppm) of lead. The Daw Tway samples also contained high arsenic levels, as high as 7,100 ppm.

Lead toxicity may be caused through fruits and vegetables contaminated with high lead levels from the soils where they were grown. The soil accumulates lead levels generally from pipes, lead paint and residual emissions from leaded gasoline that was used before the Environment Protection Agency issued the regulation around 1980. In order to prevent the general population from domestic lead poisoning, it is necessary to educate people about the major sources of lead poisoning.

Lead from water pipes coming into homes is one of the major sources (Moore, 1977). For ingested lead, the rate of absorption by the body is very high with almost 20–70% and in children it is even higher. However the rate of skin absorption for inorganic lead is low.

## Occupational exposure

Occupational exposure is a major source for lead poisoning in adults. According to estimates made by the National Institute of Occupational Safety and Health (NIOSH), more than 3 million workers in the United States are potentially exposed to lead in the workplace (Staudinger & Roth, 1998). Occupational exposure as the major concern and also the main cause of lead poisoning was reported by Needleman (2004). The common working facilities that involve lead containing products are radiation shields, ammunition, certain surgical equipment, developing dental X-ray films prior to digital X-rays, fetal monitors, plumbing, circuit boards, jet engines, and ceramic glazes (Patrick, 2006). All these increase the chances of toxicity with increasing exposure. In addition, many other occupational workers like lead miners and smelters, plumbers and fitters, car mechanicians, glass manufacturers, construction workers, battery manufacturers and recyclers, firing range instructors, and plastic manufacturers are at risk for lead exposure. Occupations like welding and manufacture of battery recycling present also a risk for lead exposure (Sanborn *et al*., 2002). Parents who are exposed to lead at workplaces generally bring lead dust to their home with clothes or on their skin, thus increasing the chances of exposure in their children (Watts, 2009). The boom of industrialisation in the modern world makes use of lead and lead products. Thus due to the industrial use of lead in modern times, the routes by which exposure generally occurs in humans is difficult to trace exactly.

Lead is a common environmental pollutant. Exposure to lead occurs mainly at occupational sites, production of lead-acid batteries or pipes, metal recycling and foundries (Woolf *et al*., 2007). Children living near such places are also at risk of elevated blood lead levels. In August of 2009, 2000 children living near zinc and manganese smelters were found to be poisoned with lead, an incident which resulted in riots (Watts, 2009). Other common things which cause lead exposure are lead in the air, household dust, soil, water, and commercial products (Rossi, 2008).

In cases of chronic exposure, lead often sequesters in the highest concentrations first in the bones then in the kidneys. According to the US Centres for Disease Control and Prevention and the World Health Organization, a blood lead level of 10 μg/dL or above is a cause for concern. However there is no threshold value below which lead exposure can be considered safe. It has been found to impair development and have harmful effects even at lower levels (Rossi, 2008; Barbosa *et al*., 2005). A variety of compounds formed by lead exists in the environment in different forms (Grant, 2009). Poisoning and its features also differ between organic and inorganic lead (Kosnett, 2007). Organic lead poisoning is now very rare around the world because of withdrawal of organic lead compounds as gasoline additives. Nevertheless, such compounds are still used in industrial settings. Organic lead compounds cross the skin and respiratory tract easily and quickly, affecting predominantly the central nervous system.

## Signs and symptoms

Lead poisoning causes a variety of symptoms, including abnormal behaviour which varies from person to person, while time of exposure plays an important role (Kosnett, 2005). There are also studies which show no symptoms of lead poisoning even with elevated levels of lead in the body (Mycyk *et al*., 2005). The question what makes such differences in the human body is an issue of major concern. Why are there such differences in attaining toxicity of lead or other toxic substances. Why for one group of people any increase in the concentration gives toxicity and for another the same level has no effect. Currently there is no evidence from the literature about such differences, however it is hoped that future research could explore this area and figure out the possible reasons. It usually takes weeks to months to build up the manifestations in the body by chronic lead exposure; however acute symptoms and signs also occur from short time intense exposures (Dart *et al*., 2004)

Organic lead is perhaps more toxic than inorganic lead because of its lipid soluble nature, which results in rapid consequences (Timbrell, 2008). However, the lead levels at which signs and symptoms appear vary widely, depending on unknown characteristics of each individual (Bellinger, 2004).

Blood lead levels from 25 and 60 μg/dL give rise to neuropsychiatric effects such as delayed reaction times, irritability, and difficulty in concentrating, as well as slowed down motor nerve conduction and headache (Merill *et al*., 2007). Anaemia may appear at blood lead levels higher than 50 μg/dL (Merill *et al*., 2007). In adults, abdominal colic, involving paroxysms of pain, may appear at blood lead levels higher than 80 μg/dL (Kosnett, 2005). High blood lead levels which exceed 100 μg/dL cause very severe manifestations, like signs of encephalopathy (condition characterized by brain swelling) accompanied by increased pressure within the skull, delirium, coma, seizures, and headache (Henritig, 2006). However such manifestations appear in children at lead levels of 70 μg/dL and more.

Central nervous system and neuromuscular manifestations usually result from intense exposure, while gastrointestinal features usually result from exposure over longer periods (Brunton *et al*., 2007). Signs and symptoms of chronic exposure include loss of short-term memory or concentration, depression, nausea, abdominal pain, loss of coordination, and numbness and tingling in the extremities (Patrick, 2006). Fatigue, problems with sleep, headaches, stupor, slurred speech, and anaemia are also found in chronic lead poisoning (Pearce, 2007). Children with chronic poisoning generally show aggressive behaviour and refuse to play.

## Effects on children

Pregnant women who have elevated blood lead levels are at a risk of premature birth or of babies with a low birth weight. The foetus may be adversely affected at blood lead concentrations well below 25 μg per deciliter (Bellinget *et al*., 1987). Blood lead levels in the neonate were found to be higher than simultaneous maternal lead levels (Shannon, 2003; Bellinger, 2005). Emaciated women with substantial exposure to lead prior to pregnancy are considered to be at increased risk.

**Figure 2:**
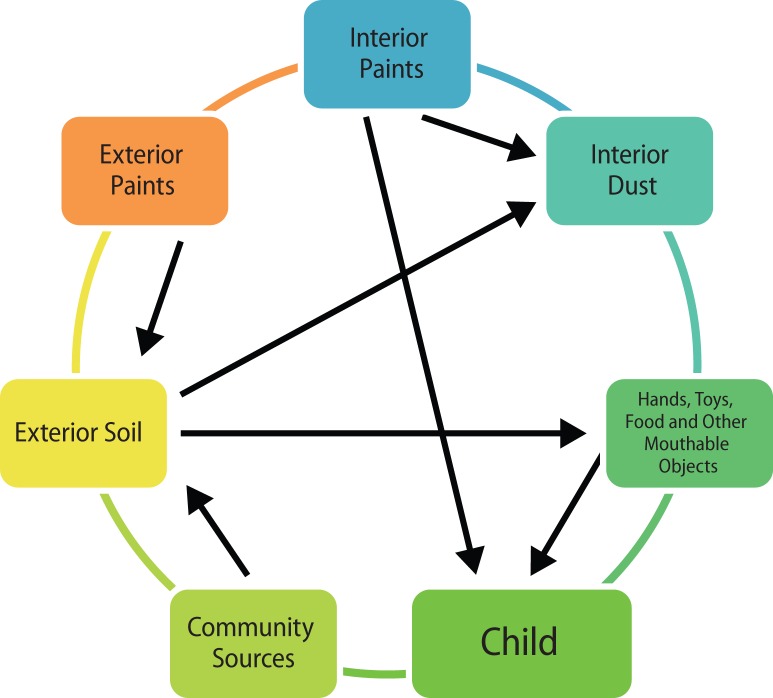
Scheme how children become easy targets for lead poisoning in the environment home.

Children have been repeatedly reported to be at higher risk for lead poisoning because their bodies are in a state of growth and development (Chisolm & Harrison, 1956). Moreover, the absorption of lead occurs more quickly in children than in adults. Children, due to their childish behaviour, are more prone to ingest and inhale dust contaminated with lead (Landrigan, 2002). The number of ways how children become easy targets for lead poisoning are illustrated in Figure 2

## Pathophysiology

Exposure occurs through various ways like inhalation, ingestion or skin contact. Direct contact of lead or lead-based compounds through the mouth, nose, eyes and through cracks of the skin may also increase lead levels. In adults, about 35–40% of inhaled lead dust is deposited in the lungs and about 95% goes into the bloodstream (Merill *et al*., 2007). On ingestion of inorganic lead almost 15% is absorbed, however this value is higher in children, pregnant women and people with deficiencies of calcium, zinc or iron (Karri *et al*., 2008). A certain amount of the lead which is generally bound in tissues like bones, teeth, hair or nails is considered to be nontoxic because of its unavailability to other tissues. The rate of absorption of lead in bones and teeth is high amounting to almost 94% in adults, while in children this rate is 70%, which allows the soft tissues to absorb more lead and thus to cause serious health consequences (Barbosa Jr *et al*., 2005). The half-life of lead in such tissues results in its induction into the blood stream long after the initial exposure (Patrick, 2006). Blood lead has a lower half-life of just about 40 days in humans. This increases in the case of pregnant women and of children whose bones are in a developing stage. The developing bones in children which undergo remodelling allow the lead to be continuously reintroduced into the blood stream (Barbosa Jr *et al*., 2005).

**Figure 3:**
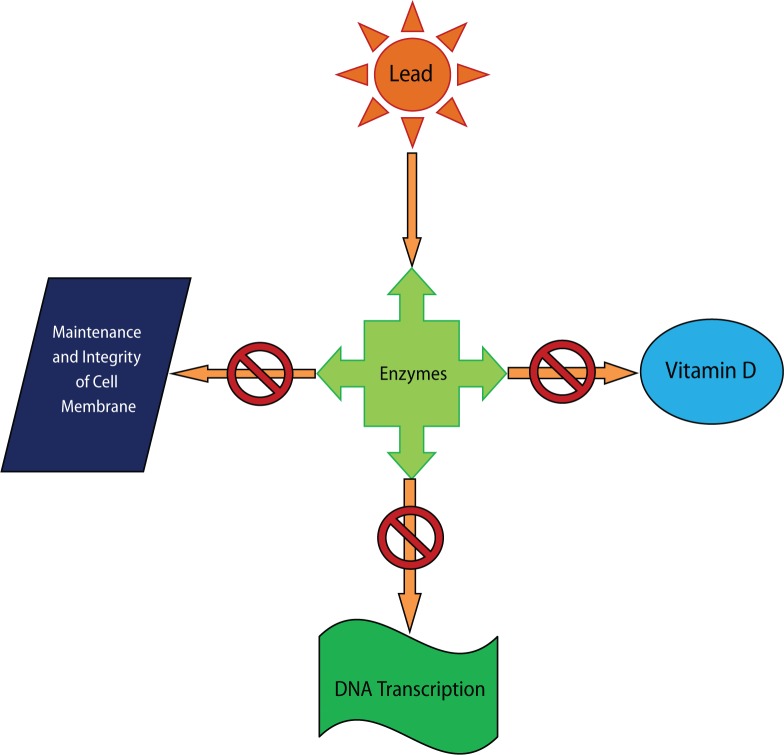
Illustration of the action of lead on enzymes, leading to the disruption of vitamin D synthesis, maintenance of cell membrane and DNA transcription.

Due to a prolonged exposure of lead for years, a much slower clearance takes place. This is due to prolonged accumulation of lead in bones released over a long period of time (Grant, 2009). Along with bones, teeth and blood, many other tissues store lead in the body, *i.e.* the brain, spleen, kidneys, liver and lungs (Dart *et al*., 2004). Small amounts of lead were found to be removed through faeces and small amounts through hair, nails and sweat (Rubin & Strayer, 2008). And yet, the interesting point to include is that lead has been found to have no physiological role in the body (Wolf *et al*., 2007; Rubin & Strayer, 2008), while its harmful effects are manifold. The effects of lead have been well studied also at cellular level. Heavy metals, including lead, create reactive radicals which damage cell structures, including DNA and cell membrane (Kosnett, 2006). Lead also interferes with the enzymes that help in the synthesis of vitamin D and with enzymes that maintain the integrity of the cell membrane. Lead was also found to interfere with DNA transcription. Illustrations are shown in Figure 3.

As lead disrupts the maintenance of the cell membrane, red blood cells with a damaged membrane become more fragile, which results in anaemia (White *et al*., 2007). Lead is also speculated to alter the permeability of blood vessels and collagen synthesis (Needlemann, 2004). Damaged activity of cells of the immune system, such as polymorphonuclear leukocytes, results in decreasing immune activity (Kosnett, 2006).

One of the main reasons for lead poisoning causing anaemia is that lead interferes with the activity of an essential enzyme called delta-aminolevulinic acid dehydratase, or ALAD, which is important in the biosynthesis of heme, the cofactor found in haemoglobin (Patrick *et al*., 2006). Heme precursors, such as aminolevulinic acid, have been found to build up due to their interference with lead, which may be directly or indirectly harmful to neurons (Fujita *et al*., 2002). In a recent finding accumulation of organic lead in the liver was found to induce oxidative imbalance and protein impairment that may result in ER stress followed by liver injuries (Fang *et al*., 2014). In another recent study the effect of lead exposure on C57BL/6J mice was observed. Early chronic low-level lead exposure yielding BLLs ranging from 1.98 to 14.84 μg/dL was found to disrupt exploratory activity in pre-adolescent mice (Flores & Sobin, 2014). In another recent study the authors studied the comorbid effects of lead exposure and high fat diet (HFD). They observed that both lead and HFD were involved in suppressing osteoblastogenesis and altered progenitor cell differentiation, promoting osteoclastogenesis and increasing adipogenesis. In addition, an increased *in vitro* PPAR-γ activity and inhibited β-catenin activity were observed in MC3T3 cells due to lead and non esterified fatty acids. They further concluded that lead and HFD were involved in producing selective deficit bone accrual in which reduced Wnt signalling may be involved with associations in progenitor cell activity alteration (Beier *et al*., 2015).

## Renal system

A number of studies found low level environmental lead exposure to be associated with accelerated deterioration of chronic renal insufficiency (Yu *et al*., 2003). Even at levels far below the limits of normal ranges in the general population, both blood lead level and blood lead burden were found to be increased, predicting accelerated progression of chronic renal disease. Wedeen *et al*. (1975) studied the effects of occupational lead hazards on patients. They observed tubular dysfunctions in patients who underwent biopsy. Such studies suggest that lead nephropathy may be an important occupational hazard. Excretion of the waste product urate was reported in lead poisoning, suggesting gout, in which urate builds up in the body (Rubin and Strayer, 2008; Ekong *et al*., 2006; Wright *et al*., 1984). Prolonged exposure to lead is associated with increase in blood pressure and some studies showed a connection between lead exposure and coronary heart disease, heart rate variability, and death from stroke, but this evidence is limited (Lin & Huang, 1994). Chronic lead nephropathy occurred due to years of lead exposure manifested in kidney biopsy by moderate focal atrophy, loss of proximal tubules and interstitial fibrosis (Benjelloun *et al*., 2007). Low level environmental lead exposure may accelerate renal insufficiency in patients without diabetes who have chronic renal disease (Lin *et al*., 2003). The authors also showed that repeated chelation therapy may improve renal function and slow down the progression of renal insufficiency.

## Reproductive system

**Figure 4:**
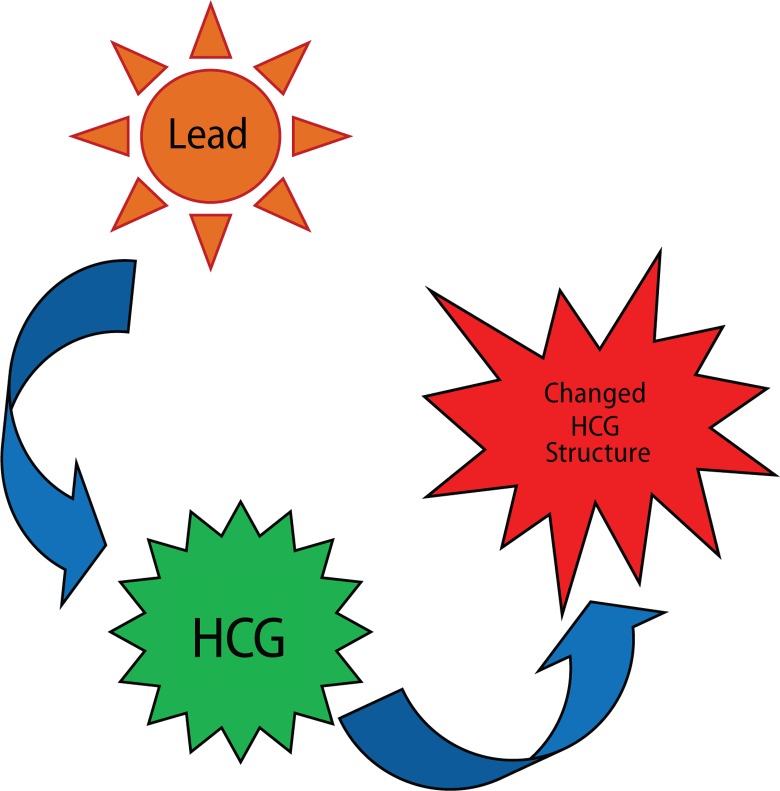
Depiction of lead changing the structure of HCG.

The reproductive system of both males and females is affected by lead. In males sperm count is reduced and other changes occur in the volume of sperm when blood lead levels exceed 40 μg/dL. Activities like motility and the general morphology of sperm are also affected at this level (Navas-Acien *et al*., 2007). The problems with the reproductivity of females due to lead exposure are more severe. Toxic levels of lead can lead to miscarriages, prematurity, low birth weight, and problems with development during childhood (Park *et al*., 2008). Blood lead levels in mothers and infants are usually similar as the lead present in mother blood passes into the foetus through the placenta and also through breast milk (Dart *et al*., 2004). As bones store the highest content of lead, if metabolic changes mobilise the lead from bones into the blood due to pregnancy, the lead toxic risks increase. However increased calcium intake during pregnancy can help mitigate this phenomenon (Grant, 2009). Low doses of lead were found to significantly reduce the number of sperms within the epididymis of mice, while high doses reduced both the sperm count and percentage of motile sperms and led to an increased percentage of epididymal abnormal sperms. Lead directly targets testicular spermatogenesis and also the sperms in the epididymis inducing reproductive toxicity (Wadi & Ahmad, 1999). In lead treated groups suppressed rates of serum testosterone, intratesticular sperm counts, and sperm production rates were reported (Apostoli *et al*., 1998; Sokol, 1989). On investigating the reversibility of the toxic effects of lead on the male reproductive axis, it was shown that serum testosterone and sperm parameters normalised at the end of the recovery period in prepubertal animals yet not in pubertal animals (Sokol, 1989). Prepubertal rats were shown to be less sensitive to the toxic effects of lead than rats whose exposure to lead was started after initiation of puberty (Sokol & Berman, 1991). In rats exposed to lead, an 80% reduction in plasma and testicular testosterone and a 32% reduction in the plasma luteinizing hormone (LH) were reported. In lead exposed rats a sharp decline in the testosterone: LH ratio was observed (Thoreux-Manlay *et al*., 1995). The detailed mechanism of how lead induces male infertility was reviewed (Mohsen *et al*., 2011). Some authors have suggested that a delay in the development of the sex specific pituitary growth hormone secretion pattern would possibly lead to growth effects of lead rather than to a persistent defect in development (Ronis *et al*., 1996). Long-term low-dose lead exposure was shown to alter the signalling system between the hypothalamus and pituitary gland of male rats. This signalling is disrupted by long-term exposure, altering thereby the gonadotropin-releasing hormone system in the male rat (Sokol *et al*., 2002). In a recent study by Zhang *et al*. (2014), the authors investigated lead interactions with human chorionic gonadotropin (HCG). Investigation of HCG by UV-vis absorption spectroscopy, circular dichroism spectroscopy, and ELISA indicated that lead acetate changed the secondary structure of HCG by loosening and destruction of the HCG skeleton and by increasing the hydrophobicity around Tyr residues, which resulted in decreased bioactivities of HCG (Figure 4). This work presents direct interactions of lead with sex hormones and suggests a possible mechanism of lead induced reproductive toxicity at molecular level.

## Nervous system

The brain is the most sensitive organ to lead exposure (Cleveland *et al*., 2008). In a child's developing brain, synapse formation is greatly affected in the cerebral cortex by lead. Lead also interferes with the development of neurochemicals, including neurotransmitters, and organisation of ion channels (Casarett *et al*., 2007). Lead poisoning also causes loss of neuron myelin sheath, reduction in the number of neurons, it interferes with neurotransmission and decreases neuronal growth (Pearson & Schonfeld, 2003). The brain of adults exposed to increased lead levels during their childhood also shows a decreased volume, especially in the prefrontal cortex on MRI (Cleveland *et al*., 2008). Lead is able to pass through the endothelial cells at the blood brain barrier because it can substitute for calcium ions and be taken up by calcium-ATPase pumps, thereby interfering with synapse formation. Children with blood lead concentration greater than 10μg/dL are at higher risk for developmental disabilities (Brunton *et al*., 2007). The effect of lead on children´s cognitive abilities takes place at very low levels (Xu *et al*., 2009; Park *et al*., 2008; Sanders *et al*., 2009). There is apparently no lower threshold to the dose-response relationship below which lead exposure is treated as safe (Meyer *et al*., 2003). Blood lead levels lower than 5 μg/dL were found to be associated with reduced academic performance (Bellinger, 2008; Needlemann *et al*., 1990). Blood lead levels below 10 μg/dL were reported to be associated with lower IQ and behaviour problems such as aggression, in proportion with the given blood lead level (Guidotti & Ragain, 2007). Between the blood lead levels of 5 and 35 μg/dL, an IQ decrease of 2–4 points for each μg/dL increase was reported in children (Brunton, 2007). Increased blood lead levels are also associated with a decrease in cognitive performance and with other psychiatric conditions like depression and anxiety (Jacobs *et al*., 2002). An increase in the blood lead levels from 50 to about 100 μg/dL in adults was found to be associated with more severe conditions, like permanent impairment of central nervous system function (Bellings *et al*., 2004). With increased lead exposure in children, an increase in neuropsychiatric disorders like attention deficit hyperactivity disorder and antisocial behaviour were found (Sanders *et al*., 2009). Prenatal and early childhood lead exposure was reported in correlation with violent crimes in adulthood (Park *et al*., 2008). The highest lead levels in the air were also shown to deviate normal behaviour and turn to become aggressive and violent, thus for example the highest murder rates were found in countries with high levels of lead in the air (Neelemann *et al*., 2004). One study theorizes that lead exposure explains 65% to 90% of the variation in violent crime rates in the US (Shih *et al*., 2007; Kosnett *et al*., 2007). Another study showed a strong association between preschool blood lead levels and subsequent crime rate trends over several decades across nine countries (Nevin, 2007).

The hippocampus is a part of the brain involved in learning and memory. The main reasons for lead interfering with learning particularly in children is that it damages the cells within the hippocampus. In rats exposed to lead, structural damage such as irregular nuclei and denaturation of myelin were reported (Mycyk *et al*., 2005).

Lead is also involved in interfering with the release of neurotransmitters (Dart *et al*., 2004; Needlemann, 2004). Neurotransmitters are chemicals used by neurons to send signals to other cells. This interference leads to the disruption of communication between cells. Lead usually interferes with the neurotransmitter glutamate which is important for many functions, like learning. It operates by blocking NMDA receptors. Blocking of such receptors is thought to be the main target of lead toxicity. One study found that in addition to inhibition of the NMDA receptor, lead exposure also decreased the amount of gene for this receptor in part of the brain (Kosnett, 2005). Lead was also found to be involved in apoptosis of brain cells in animal studies (Nedlemann, 2004).

## Diagnosis

In order to prevent lead poisoning and toxicity, proper diagnosis is a primary and rather important issue. In order to make a proper diagnosis, an inquiry about the possible routes of exposure is a must (Nevin, 2007). The inquiry should include medical history and determination of clinical signs. The involvement of proper staff, *i.e.* clinical toxicologists and medical specialists, can help in establishing proper diagnosis and treatment.

Basophilic stripping is an important sign of lead poisoning. This stripping makes dots in red blood cells visible through the microscope (Patrick, 2006). Thus an examination of blood film for such signs could be effective in detecting lead poisoning. Lead poisoning is associated with iron deficiency anaemia. Lead poisoning can also be evaluated by measuring erythrocyte protoporphyrin (EP) in blood samples (Patrick, 2006). EP is known to increase when the amount of lead in the blood is high, with a delay of a few weeks (Kosnett, 2007). However, the EP level alone is not sensitive enough to identify elevated blood lead levels below approximately 35μg/dL (Patrick, 2006). Due to this higher threshold for detection and the fact that EP levels also increase in iron deficiency, the use of this method for detecting lead exposure has decreased. Blood lead levels are an indicator mainly of recent or current lead exposure, not of the total body burden. The measurement of blood lead level does not give the actual account of lead stored in the body, it is just an indicator of recent lead exposure. Whole body lead can be measured in bones noninvasively by X-ray fluorescence; this may be the best measure of cumulative exposure and total body burden (Kosnett, 2006). X-rays may also reveal lead-containing foreign materials such as paint chips in the gastrointestinal tract (Kosnett, 2007; Grant, 2009).

## Prevention and treatment

Lead poisoning causes severe effects and is a matter of serious concern, yet importantly, it is preventable. The best approach is to avoid exposure to lead (Rossi, 2008). It is recommended to frequently wash the children´s hands and also to increase their intake of calcium and iron. It is also recommended to discourage children from putting their hands, which can be contaminated, in their mouth habitually, thus increasing the chances of getting poisoned by lead. Vacuuming frequently and eliminating the use and or presence of lead containing objects like blinds and jewellery in the house can also help to prevent exposures. House pipes containing lead or plumbing solder fitted in old houses should be replaced to avoid lead contamination through drinking water. It is believed that hot water contains higher lead levels than does cold water, so it is recommended that for household uses cold water should be preferred to hot water (Baselt & Randall, 2008).

The treatment for lead poisoning consists of dimercaprol and succimer (Park *et al*., 2008). Due to the persistent findings on cognitive deficits caused by lead poisoning particularly in children, widespread reduction of exposure should be mandatory.

Lead poisoning is generally treated by using chelating salt disodium calcium edentate, which is the calcium chelate of the disodium salt of ethylene-diamine-tetracetic acid (EDTA). Such chelating agents have a great affinity to the removing agent. The chelating agent for lead has a greater affinity to lead than calcium and so the lead chelate is formed by exchange. This is then excreted in urine, leaving behind harmless calcium. Blood lead levels were shown to be lowered by treatment with succimer used as chelation therapy in children exposed to lead to improve their neuropsychological development. And yet, though succimer was observed to help in reducing blood lead levels, it failed in improving the scores of cognition tests (Rogan *et al*., 2001). There is a number of antioxidants which are believed to act against toxicity of chemicals like lead and its related compounds. A new technique called nano-encapsulation of antioxidants may provide improved biodistribution and bioavailability of poorly soluble therapeutics through solubilisation (Flora *et al*., 2012). Encapsulation of curcumin in a pluronic block copolymer demonstrated a slow and sustained release of curcumin and showed anticancer activity comparable with free curcumin (Sahu *et al*., 2010). These new techniques may hold a promise for treating a number of human diseases. In a very recent study, it was observed that puerarin promoted Akt and GSK-3β phosphorylation in PC12 cells exposed to lead acetate. The authors of the study concluded that puerarin as a phytoestrogen might be an attractive agent for prevention and treatment of chronic diseases related to lead neurotoxicity. In another recent finding beta-carotene was observed to have an antioxidant action and exert some beneficial effects in lead poisoning, independent of chelation (Dobrkowski *et al*., 2014). The authors also found significantly decreased homocysteine levels due to administration of betacarotene in lead exposed workers. Recently a study on a group of workers occupationally exposed to lead found that those treated with N-acetylcysteine (NAC) showed a significant reduction in their blood lead levels. In addition, all groups receiving NAC were shown to have significantly elevated activity of glutamate dehydrogenase. It was further reported that treatment with NAC normalised the level of homocysteine and decreased oxidative stress. It was thus concluded that NAC could be recommended as an alternative therapy for chronic lead toxicity in humans (Kasperczyk *et al*., 2015).

## Conclusion

Of all the heavy metal poisonings, lead poisoning appears to be rather prominent. The use of lead has been evidenced from ancient times and its toxicity reports are well documented. Due to its important physico-chemical properties, it has been used all over the world. With the onset on industrialisation from the seventeenth century onwards, its use increased manifold, leading to increased toxicity in humans. Children are at a higher risk, particularly at sites where lead related occupations are nearby their playing grounds. Workers who are occupationally exposed to lead are also at increased risk of lead poisoning. Children of parents who are occupationally exposed to lead should be frequently checked for lead levels in their blood to avoid lead related risks. Lead toxicity is evident from the literature and there is almost no function in the body which is not affected by lead. Lead toxicity disrupts the functions of the digestive system, nervous system, respiratory system, reproductive system, *etc.* In addition, lead prevents enzymes from performing their normal activities. Lead even disrupts the normal DNA transcription process and causes disability in bones. Lead as such has no physiological role in the body and even smaller levels of lead can cause toxicity. The good news is however that it can be reversed and the levels of lead can be reduced from the body by a number of techniques used nowadays. The prominent ones among them are chelation therapy, nano-encapsulation, N-acetylcysteine (NAC). A number of antioxidants also help in the removal of lead from the body. Though there are several methods of treatment available nowadays, it is certainly better to prevent direct exposure to toxins and thus preclude future consequences. It is also recommended that parents should educate their children about how to prevent accidental lead poisoning. The treatment strategies are not equally effective for everybody due to the differences ranging from genetic factors to environment and diet.
